# Splicing modulators: on the way from nature to clinic

**DOI:** 10.1038/s41429-021-00450-1

**Published:** 2021-08-03

**Authors:** Tilman Schneider-Poetsch, Jagat Krishna Chhipi-Shrestha, Minoru Yoshida

**Affiliations:** 1 Chemical Genomics Research Group, RIKEN Center for Sustainable Resource Science, Wako, Saitama Japan; 2 Department of Biotechnology, Graduate School of Agricultural and Life Sciences, The University of Tokyo, Bunkyo-ku, Tokyo Japan; 3 Collaborative Research Institute for Innovative Microbiology, The University of Tokyo, Bunkyo-ku, Tokyo Japan

**Keywords:** Enzyme mechanisms, High-throughput screening, Target identification, Natural product synthesis

## Abstract

Over the course of more than two decades, natural products isolated from various microorganisms and plants have built the foundation for chemical biology research into the mechanism of pre-mRNA splicing. Hand in hand with advances in scientific methodology small molecule splicing modulators have become powerful tools for investigating, not just the splicing mechanism, but also the cellular effect of altered mRNA processing. Based on thorough structure-activity studies, synthetic analogues have moved on from scientific tool compounds to experimental drugs. With current advances in drug discovery methodology and new means of attacking targets previously thought undruggable, we can expect further advances in both research and therapeutics based on small molecule splicing modulators.

## Introduction

Even with advances in scaffold-based synthesis and natural product-like molecule production, genuine natural products still form a corner stone of chemical biology. However, the path from identification of a new and interesting molecule to a detailed understanding of its mechanism of action, cellular effect and utility in research and in medicine is a long, winding and arduous one. Many compounds prove to act upon different targets than initially thought. Discovery of practical uses as bioprobes or, with a lot of good luck, as therapeutics may take many years. Splicing inhibitors are no exception. While the first molecules were described in 1992, it took till 2007 before their mechanism came to light and only now are we understanding their profound effect on cellular mRNA metabolism, signaling and physiology. Recent studies are testament to the utility of natural products, the tenacity of researchers working on them, as well as significant advances in research technology and methodology.

### Initial discoveries

**Fig. 1:**
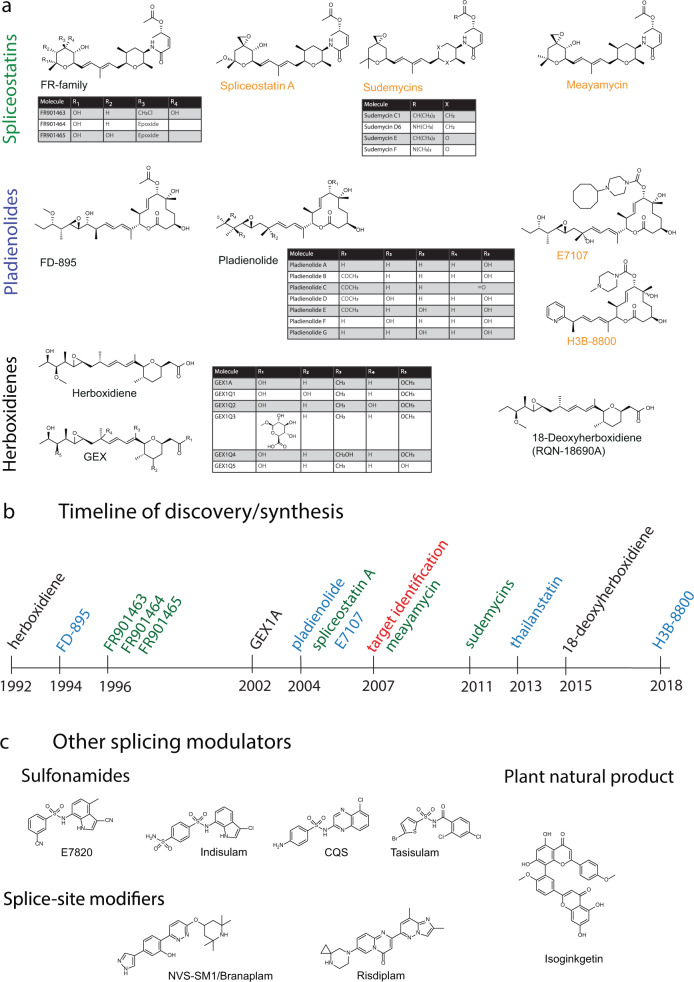
**a** The three families of SF3B1-targeting splicing modulators; spliceostatins, pladienolides and herboxidienes. Synthetic and semisynthetic derivates of the original natural products are labeled in orange. **b** Timeline of discovery or synthesis of the molecules shown in (**a**). Herboxidienes are labeled in black, spliceostatins in green and pladienolides in blue. **c** Splicing modulators not targeting SF3B1

In 1992, herboxidiene, a *Streptomyces chromofuscus* natural product with strong phytotoxic activity was first described [1, 2] (Fig. 1a, b). It was re-discovered in a different strain of *Streptomyces* in 2002, this time as one of a group of molecules named GEX1 compounds [3, 4], displaying cytotoxicity in the low to mid-nanomolar range against human tumor cell lines. At the time closer mechanistic studies were not undertaken and the molecular target remained unknown.

Four years later, evaluating whether transcriptional modulators would have antitumor activity, three molecules isolated from a broth of *Pseudomonas sp.* No.2663. were tested for their effect on the viral SV-40 promoter [5, 6]. The compounds, labeled FR901463, FR901464, and FR901465 led to significant promoter activation. The molecules were cytotoxic at low nanomolar concentration and inhibited the cell cycle in G1 and G2/M phase. FR901464 displayed strong antitumor activity, extending the lifespan of mice carrying the P388 lymphoma cell line and also inhibiting the growth A549 human lung adenoma xenografts. Observing internucleosomal DNA fragmentation at higher concentration, the authors assumed the FR compounds to act directly on chromatin, thereby changing transcriptional activity. Spliceostatin A, a methyl ketal derivative of FR901464 proved more stable in solution at equal potency and became pivotal in identifying the FR compounds’ mechanism of action [7]. Consequently, this family of molecules became known as the spliceostatins.

In 1994, another report appeared in this journal, presenting a cytotoxic polyketide from a culture of *Streptomyces hygroscopicus strain* A-9561, isolated in Okinawa [8]. The molecule, named FD-895 proved toxic to doxorubicin-resistant HL-60 leukemia cells, but again mechanistic studies did not commence. Interest in FD-895 reemerged when the structurally related pladienolide family of natural products attracted attention a decade later. Pladienolides were first described in 2004 when a group of 7 related molecules was isolated from a *Streptomyces platensis* broth, taken from a soil sample from Kanagawa prefecture, Japan [9–11]. The initial study aimed at finding new antitumor agents, specifically compounds that would prevent angiogenesis in the hypoxic environment of solid tumors. The screen utilized an alkaline phosphatase reporter driven by the hypoxia inducible factor 1α (HIF1α)-dependent promoter of vascular endothelial growth factor (VEGF) [9, 11].

While it appeared that the active pladienolides did inhibit VEGF expression more strongly than they proved cytotoxic, the IC_50_ values for both activities only differed around 2–4 fold [9, 11]. Therefore, a specific mechanism targeting HIF1α signaling appeared unlikely. The paper offered a limited amount of structure activity relationship data and noted that pladienolide B (PlaB) inhibited growth of several tumor cell cultures from a panel of 39 cancer cell lines at low nanomolar IC_50_ values, even cell lines resistant to clinical anticancer agents, such as etoposide, cisplatin, camptothecin, vincristine, taxol and 5-flurouracil. PlaB did not appear to kill cells indiscriminately but was only specifically cytotoxic to some cell lines in the panel, though it remained unclear what drove its selectivity. In mouse xenograft models, PlaB proved effective against tumors from the BSY-1 breast cancer cell line [10]. These initial experiments indicated that pladienolides likely acted by a novel mechanism of action but gave little indication of the molecules’ actual target.

In addition, FD-895 and pladienolides share some superficial structural similarity with the lactimidomycin and isomigrastatin family of translation inhibitors, showing once more that 2D molecular structures are not predictive of activity [12, 13].

Since the herboxidienes, spliceostatins and pladienolides were each discovered assessing completely unrelated activities, nobody would have suspected that, despite representing vastly different structures, the three compounds share virtually identical mechanisms of action. In light of their activity and potency against tumor cells, synthetic routes to producing FR901464 and pladienolide were developed, though owing to an inordinate number of necessary steps, total synthesis for clinical use appeared impractical [14–17]. The spectrum of available active compounds keeps also increasing by natural means and for a change not from another strain of *Streptomyces*. The proteobacterium *Burkholderia thailandensis* MSMB43 yielded thailanstatin A [18, 19]. Not as potent as FR901464 itself, thailanstatin A still inhibits tumor cell growth in the mid to high nanomolar range [20]. In a similar manner, a herboxidiene derivative, RQN-18690A or 18-deoxyherboxidiene, was re-discovered in a screen for angiogenesis inhibitors [21]. It’s activity against human umbilical vein endothelial cells could also be traced back to SF3B1 inhibition.

Despite structural dissimilarity, computational studies indicated that the solution structures of both FR901464 and PlaB adopt similar conformations, centered on their central diene moiety, suggesting that they could bind the same physiological target [22]. Based on this hypothesis fully synthetic molecules centered on a common pharmacophore between FR901464 and PlaB were produced. The analogue displayed cytotoxic activity and induced G2/M cell cycle arrest, but not as potently as the original natural products. Since FR901464 was difficult to synthesize and somewhat unstable in solution, strategies were developed to generate simplified and stable analogues, culminating in the sudemycin family of molecules [23], which also inhibited tumor cell growth, albeit at IC_50_ values in the micromolar range [24–26].

While sudemycins suffered from significantly decreased potency, another synthetic attempt at producing FR901464 analogues yielded meayamycin, which, if anything, proved even more potent than the original natural product [17]. As can be seen in Fig. 1, the chemical space occupied by splicing modulators appears rather accommodating. It took till 2007 before the converging mechanisms of all molecules describes started to become apparent, with PlaB and SSA becoming the first known specific inhibitors of pre-mRNA splicing [27, 28].

### Target identification

**Fig. 2:**
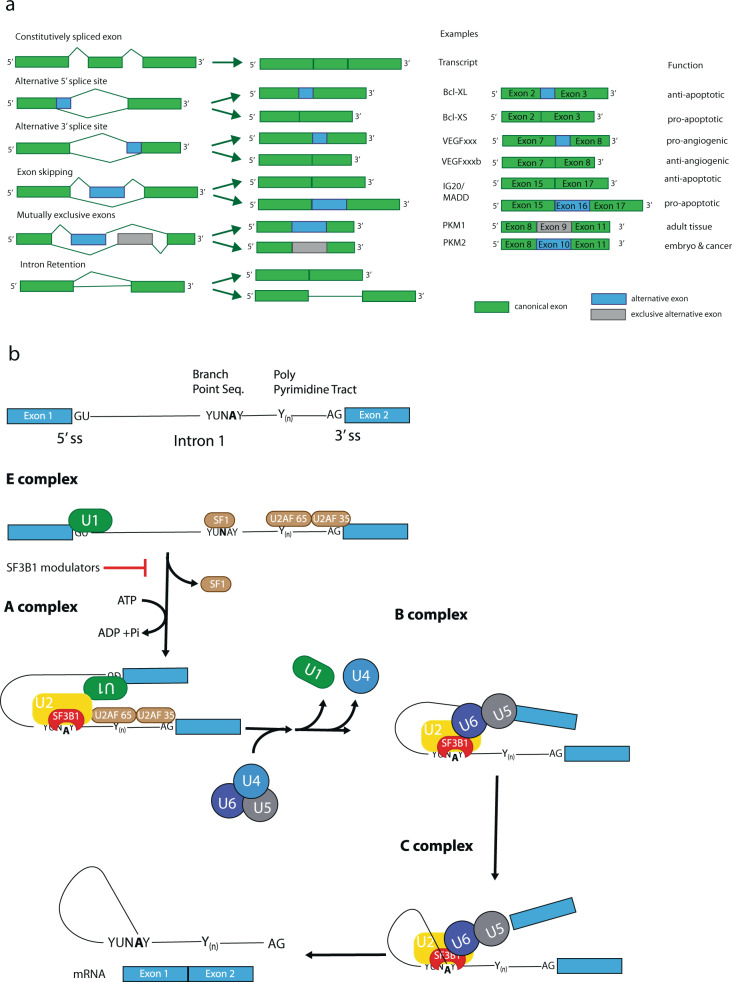
**a** Types of alternative splicing events with illustrative examples. Green denotes canonical exons, while included alternative splice sites or exons are indicated in blue, mutually exclusive alternative exons in gray [31]. **b** Simplified overview of spliceosome formation and pre-mRNA splicing

Splicing, the process of removing intronic sequences from a primary transcript, constitutes a key step in gene regulation of eukaryotic cells (reviewed in [29]). The coordinated interplay of several large ribonucleoprotein complexes not only allows producing a readable template for protein synthesis, likely it presents the very key to the complexity of large, multicellular organisms [30]. Every splicing reaction involves a coordinated intramolecular transesterification of an RNA polynucleotide, excising an internal sequence stretch. Via splicing the same primary RNA transcript can yield several different protein isoforms specific to a particular cell type, tissue or developmental stage. This goes so far, that splice isoforms of the same transcript may produce proteins with antagonistic properties (reviewed in [31]). Besides simply cutting out an intronic sequence, splicing of one pre-mRNA may lead to different outcomes. These include the choosing of alternative 3ʹ and 5ʹ splice sites, exon skipping (ES), inclusion of a cassette exon or of mutually exclusive exons (Fig. 2a). In general, an intron is defined by its 5ʹ and 3ʹ splice sites. Toward the 3ʹ end lies the branchpoint sequence containing the adenosine residue whose 2’OH group will conduct the nucleophilic attack on the 5ʹ splice site’s phosphodiester bond, creating a free 3ʹ hydroxyl group, which can then perform an attack on the 3ʹ splice site, connecting two exons and excising a lariat shaped intron (Fig. 2b). This branchpoint A (BPA) is surrounded by the branch-point sequence (BPS) and followed by a poly-pyrimidine tract. Naturally, choosing the right splice site and branch point requires a high degree of regulation and control [29]. Besides the core spliceosome, hundreds of accessory factors, including SR (serine and arginine-rich) proteins, may act as splicing enhancers or repressors, influencing the utilized splice site.

The process begins with E complex formation, where the U1 snRNP marks the 5ʹ splice site, while accessory factors SF1 and the dimeric U2AF bind toward the 3ʹ end, preparing arrival of the U2 snRNP. The U2 snRNP binds to the branch point sequence, displacing SF1, thereby forming A complex. During A complex formation the BPS will form a stable helix with the branchpoint interacting stem loop (BSL) of the U2 snRNA. The BPS is moderately conserved and the degree of complementarity to the U2 snRNA, especially within the BSL, determines the strength of the splice site, with better matches accounting for higher strength. At this point, the pre-mRNA is still intact. The actual cleavage reaction requires binding of the U4/U5/U6 tri-snRNP complex and displacement of U1 and U4. This review will focus on A complex formation, as SSA, PlaB, the herboxidienes and all their derivatives act on this step, specifically on the SF3b subcomplex of the U2 snRNP [32].

The U2 snRNP consists of the U2 snRNA polynucleotide, whose base-pairing to the branch point sequence maintains specificity, and a set of two protein subcomplexes SF3a and SF3b, as well as a set of Sm proteins which form a ring structure around the nascent transcript and enhance splicing fidelity [33]. The SF3b subcomplex, at its core consists of the proteins SF3B1 through SF3B6, PHF5A, TAT-SF1 and the ATPase PRP5. The largest SF3B subunit, SF3B1, contains two N-terminal domains for interaction with U2AF and PHF5A, followed by 20 tandem HEAT (Huntingtin, elongation factor 3, protein phosphatase 2A, target of rapamycin) repeats [34]. SF3B1 forms a long, superhelical structure, curving around PHF5A and contacting part of the U2 snRNA and many key protein factors, including PRP5 and TAT-SF1. Currently, it is thought that U2 finds the right position on the intron by interaction with U1, SF1, and U2AF. At that point the U2 BSL seems protected by TAT-SF1 and part of PRP5. RNA binding leads to PRP5 activation and the ATP-dependent displacement of TAT-SF1, such that BPS and U2 BSL can begin base-pairing, eventually disrupting the stem loop and forming an RNA-RNA double helix between U2 and pre-mRNA. In the early stages of A complex formation SF3B1 is present in an open conformation but will clamp down onto the U2-mRNA helix adopting a closed conformation, thereby also displacing PRP5. In addition, SF3B1 and PHF5A together form a pocket to protect the reactive BPA [35]. Mutations in SF3B1 have been implicated in myelodysplastic syndromes (MDS), chronic lymphocytic leukemia (CLL), chronic myeloid leukemia (CML) and a number of late-stage cancers. Mutations appear to primarily cluster around the more N-terminal HEAT repeats with changes in R625, K666 and K700 leading to alternative BPS usage and cryptic 3′ splice site selection [36–39]. Especially HEAT repeat 6 and surroundings appear as mutational hotspots, likely affecting PRP5 binding and function, thereby reducing the fidelity of branch point selection.

Since 2007 further characterization of SF3B1’s prominent role in splicing has become possible thanks to specific chemical probes. Derivatives of FR901464 and pladienolide finally allowed target identification [27, 28]. A biotinylated version of SSA, as well as derivates of PlaB incorporating radiolabels, fluorescent markers and photo-crosslinkers enabled identifying the SF3B complex as the true molecular target. Further experimentation confirmed that SSA, PlaB, herboxidienes and related compounds did interrupt pre-mRNA splicing [40, 41]. In in vitro studies the U2 snRNP interacted more weakly with the pre-mRNA in presence of inhibitor and proved prone to bind at cryptic splice sites. Furthermore, inhibition appeared to occur before ATP hydrolysis, which means that A complex formation was not completed.

**Fig. 3:**
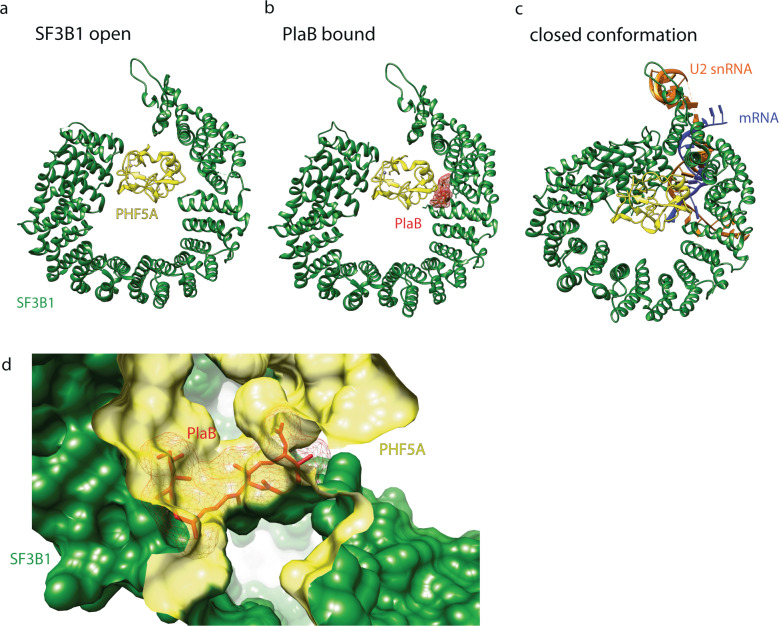
**a** Structure of SF3B1 in open conformation. **b** PlaB arrests SF3B1 in its open conformation. **c** SF3B1 in closed conformation. The closed conformation in (**c**) is based on structural data from B complex SF3B1, but is thought to be highly similar to its shape in A complex. SF3B1 is represented in green, PHF5A in yellow, PlaB in red, pre-mRNA in blue and U2 snRNA in orange. **d** Depiction of the splicing modulator binding site. Pladienolide (red) binds at the interface of SF3B1 (green) and PHF5A (yellow). Central element to the binding site is the diene moiety in the middle of PlaB (and also of spliceostatin and herboxidiene), fitting through a narrow passage between the two binding proteins. Structural information taken from PDB 5IFE (open), 6EN4 (PlaB-bound) and 6FF4 (closed conformation) [35, 44, 120]

It was soon appreciated that in vivo these molecules do not necessarily interrupt splicing in total but modulate cellular splicing behavior. On a global scale, rather than exclusively causing intron retention, a large percentage of aberrant splicing events leads to ES with a smaller fraction displaying altered 3ʹ and 5ʹ splice sites or alternative incorporation of otherwise excluded exons. Percentages differ, depending on experimental conditions and small molecule used, but it appears that intronic GC content affects the choice between ES and intron retention [42, 43]. Resistance mutations to the modulators cluster to HEAT repeats 15 and 16 on SF3B1 and also Y36 on PHF5A. The resistance mutations against the splice modulators on SF3B1 fell much further toward the C-terminus than the mutational hotspots associated with disease phenotypes. Structural studies confirmed the resistance mutations to lie around the actual drug binding site (Fig. 3) [42]. While structural data is currently limited to PlaB, the observed hourglass-shaped binding site with the diene moiety in its “neck” agrees with the idea of a common pharmacophore between the different molecular families (Fig. 3d). Drug binding to SF3B1 arrests the complex in its open conformation, thereby preventing completion of A complex formation [44] (Fig. 3a–c). While initial studies, based on the location of resistance mutations, suggested that the splicing modulators competed with the BPA, in light of structural evidence, it appears more likely that the molecules compete with the intron for the open conformation of the complex. This agrees with in vitro observations that once A complex formation is complete, PlaB loses its effect [45, 46].

While for all practical purposes, SSA, PlaB, herboxidienes and synthetic colleagues employ the same molecular mechanism, their cellular effects are not necessarily identical. Weaker binders, such as sudemycin D6 or herboxidiene tend to mainly induce ES but less intron retention compared to the more potent SSA or PlaB [42]. This again, appears to relate to intronic GC content with a lower GC percentage making the splice site more resistant to modulator activity compared to introns with high GC content. These observations strengthen the idea that not only the mechanism of action, but also relative potency of the binding molecule determine the efficacy of a particular modulator.

### Honorable mention

While pladienolide and SSA’s effect on splicing were serendipitous discoveries, isoginkgetin not only stands out from the molecules discussed thus far as a plant natural product, isolated from leaf extracts of *Ginkgo biloba* [47], it is also the first actively sought splicing inhibitor (Fig. 1c). Using a reporter with the luciferase coding sequence sitting downstream of a canonical splice site, isoginkgetin emerged from screening a chemical library. While thought to prevent binding of the U4/U5/U6 tri-snRNP to A complex, its mechanism has not been described in as much detail as the SF3B1-targeting compounds, likely also owing to much weaker activity. To this day its binding site remains unknown [48].

Recent studies suggest that isoginkgetin not only inhibits the proteasome [49] but also transcriptional elongation, which may indicate that its cellular effect is much messier than originally anticipated [50]. Especially the latter issue may be difficult to resolve. The original study showed stalled B complex formation in vitro requiring no *de novo* pre-mRNA synthesis, while the work on transcriptional elongation relied on directional transcriptome analysis. It remains possible that the different behavior on RNA polymerase observed between SF3B targeting molecules and isoginkgetin reflects differences in mechanism with isoginkgetin blocking splicing at a later step, producing a different cellular phenotype. A more recent study on hinokiflavone, a different plant flavonoid, reported inhibition of the SENP de-SUMOylation enzyme, albeit at fairly high concentrations. While detailed mechanistic data is still lacking, the report suggested that hyper-SUMOylation of splicing factors may interfere with proper B complex assembly [51].

### Cellular consequences

Since splicing constitutes a fundamental regulatory step at the upstream end of gene expression the downstream cellular consequences of splicing manipulation are numerous. Many different aspects of cellular dysregulation brought about by splicing modulation may each contribute to the observed anticancer activity of these molecules (Table 1).

**Table 1:** Possible antitumor mechanisms of splicing modulators

Effect	Target	Molecules	References
Cell cycle inhibition through dominant negative p27*	SF3B1	SSA	[27, 31, 73]
Changing cellular signaling and triggering cellular stress response	SF3B1	SSA	[27, 55]
Synthetic lethality in combination with SF3B1 mutations	SF3B1	H3B-8800	[76]
Changing alternative splicing of tumor-related genes, favoring tumor suppressive isoforms	SF3B1, RBM39	SSA, Indisulam, CQS, tasisulam	[74, 83, 103, 104]
Triggering antiviral response	SF3B1	H3B-8800, sudemycin D6	[70]
Generating neo-immunogens	Spliceosome	Isoginkgetin	[90, 91, 94]
Changing lncRNA behavior	SF3B1	SSA	[60]
Slowing RNA polymerase II	SF3B1	SSA	[56]
Targeting splicing factor for degradation	RBM39	Indisulam, CQS, tasisulam, E7820	[103, 104]

Besides changing splicing patterns and causing intron retention, changed splicing behavior also leads to alterations in RNA production itself [52, 53]. Genome wide transcription decreased ~20–30% upon SSA treatment [54]. Owing to altered splicing of some effector proteins such as IκBα, ERK and NF-κB signaling pathways displayed increased activity [27, 55]. Furthermore, splicing perturbation leads to slowed RNA polymerase II (RNAPII) progression with the enzyme increasingly failing to reach the end of its transcripts and concomitant decrease in Ser-2 phosphorylation on the RNAPII C-terminal domain [56, 57].

It is important to keep in mind that the snRNPs are not present in stoichiometric proportions, but that U1 concentration far exceeds the level of U2 or U4/U5/U6. Besides marking the 5ʹ splice site, U1 protects mRNA from premature cleavage and poly-adenylation [58, 59]. A recent study observed that SSA treatment led to aberrant poly-adenylation in the MALAT1 (Metastasis Associated Lung Adenoma Transcript 1), a long non-coding RNA (lncRNA), as well as a few canonical mRNA transcripts [60]. MALAT1 is the most abundantly expressed lncRNA in nuclear speckles [61]. Its elevated expression is commonly observed in metastasis-prone tumors [62]. MALAT1 plays an important role in regulating transcription, alternative splicing and posttranscriptional processing [63]. It is known to bind a number of splicing factors, including SRSF1 and hnRNPC, modulating their activities [64, 65] Under normal circumstances MALAT1 is strictly located in the nucleus, but under SSA treatment its polyadenylated form is exported to the cytoplasm. While the currently known splicing modulators do not directly alter U1 activity, impeded U2 function and the subsequent buildup of improperly processed transcripts seem to eventually exhaust the pool of available U1 snRNP and cause further cellular effects from an unexpected source. Importantly, aberrant poly-adenylation was only observed in presence of nonfunctional U2 snRNP, but not when U2 itself was depleted. The acronym MALAT1’s meaning indicates the transcript’s association with cancer [66–68]. Whether its aberrant poly-adenylation and export to the cytosol has any bearing on the antitumor activity of splicing modulators or whether it presents a phenomenon of limited scope and relevance remains to be seen, especially when considering that only a fraction of MALAT1 undergoes poly-adenylation.

The sheer amount of misspliced RNAs appears to overwhelm quality control mechanisms, including nonsense-mediated decay. Unspliced pre-mRNA accumulates in the nucleus, with nuclear speckles also increasing in size [27, 69]. Some improperly processed transcripts escape to the cytoplasm and are translated into protein. Initial studies observed production of truncated polypeptides, including a shortened, constitutively active version of the cell cycle regulator p27, labeled p27* [70–72]. p27* production and subsequent G1 cell cycle arrest likely account for part of the observed antitumor effect, though it does not explain the full extent of specific toxicity toward some cell lines [73].

Specificity likely derives from a particular tumor’s transcriptome and splicing state. Beyond SF3B1 itself many other splicing factor mutations have been implicated in hematopoietic malignancies. U2AF, SRSF1 and 2 or RBM39 may contain mutually exclusive point mutations on specific residues affecting splice site selection [74, 75]. Cancer cells bearing splicing factor mutations appear particularly sensitive to splicing modulators, with two perturbations in the same cellular system proving synthetic lethal [75–79].

Splice isoforms of VEGF may stimulate or inhibit angiogenesis, depending on whether exon 8 is spliced at a proximal (pro-angiogenic) or a distal (anti-angiogenic) splice site [80–82]. SSA treatment leads to a decrease in VEGF expression in malignant tumors, another aspect that may explain part of the antitumor activity of splicing modulators [54].

In a similar vein, CLL relies on high levels of the Bcl2 family apoptosis regulator Mcl-1 for survival. Mcl-1’s long splice isoform (Mcl-1L) has antiapoptotic activity, while the short variant (Mcl-1S) is pro-apoptotic. Splicing modulators appear to favor production of the short isoform [83, 84].

Yet another aspect in the relation between splicing and cancer has garnered attention in recent years, as myc-dependent tumors appear particularly sensitive to splicing modulators. One link connects the arginine methyl transferase PRMT5, which acts on the Sm proteins [85]. It appears that Sm protein assembly aids in the splicing of introns with weak 5ʹ splice sites, leading to increased ES. In addition, myc also drives expression of splicing factor SRSF1, an oncogenic protein in its own right [86, 87].

Some myc-driven tumors seem particularly sensitive to small molecule splicing inhibitors, possibly because the small molecule shifts an already precarious balance in splicing toward acute cytotoxicity [70, 85, 88, 89].

Perhaps focusing on isolated cells in tissue culture alone is not sufficient to appreciate the physiological role and clinical potential of splicing modulators. A recent study observed formation of double-stranded RNA species from intronic sequences upon treatment with sudemycin D6 or the more recently described synthetic PlaB derivative H3B-8800 in myc-driven triple-negative breast cancer [70]. These double-stranded RNAs appear to trigger cellular antiviral immune responses and subsequent cell death.

With mutated splicing factors, leading to altered 5ʹ and 3ʹ splice sites, proteome composition significantly changes, potentially providing a generous source of neoantigens [90–92]. A study of 32 TCGA cancer types from 8705 patients investigated the effect of alternative splicing on cancer-specific markers presented on cellular MHC-I antigen presenting complexes [91]. It found a number of tumor-specific markers absent in normal cells, which may act as neoantigens and allow development of mRNA vaccines or other immunotherapies [93]. Splicing modulation very likely alters proteome content and therefore also presented antigens, providing a potential source for immunotherapy. Data generated with isoginkgetin indicates that splicing modulation does lead to presentation of neoantigens on MHC-I complexes [94].

It was appreciated early on that splicing modulators might not only prove potent bioprobes in the laboratory but also provide clinical benefits, especially in cancer treatment. The use of splicing modulators in treating malignancies might be a case of needing to find a suitable disease for the right medication.

### Clinical trials

The first splicing modulator to enter the clinic was pladienolide’s semisynthetic derivative E7107. The molecule’s development preceded the identification of its molecular target [95] and E7107 initially showed promise in xenograft models, displaying a wide therapeutic window. Human trials against solid tumors were halted owing to dose-limiting toxicity but showed little or no clinical benefit [96, 97], though splicing modulation was observed.

Given that splicing modulators appear most efficacious against hematopoietic cancers, one could argue that the initial E7107 trials were using the right medication on the wrong disease. While research on E7107 seems to continue, it appears that focus is shifting to a different compound [98].

A more recently published synthetic pladienolide derivative may hold more promise. H3B-8800 is orally bioavailable and appears efficacious against tumors with splicing factor mutations, not limited to SF3B1 but also encompassing U2AF and SRSF2 [76]. The study reiterated differences in physiological behavior even between two related molecules with identical mechanisms of action. H3B-8800 and E7107 displayed different behavior in cell killing and splicing modulation with H3B-8800 preferentially retaining short (<300 bp) GC-rich introns with a weak BPS. These preferences did not appear as pronounced in the experimental system when using E7107. The authors argued that H3B-8800’s selectivity for short GC-rich introns especially impacted expression of other splicing regulators, including U2AF, thereby further disrupting proper mRNA processing.

Initial results from phase I clinical trials against MDS, AML and CML indicated that H3B-8800 acts on its target at similar doses as in preclinical xenograft studies [99] and leads to altered mRNA splicing. However, while the drug appeared to act as expected, clinical responses to splicing modulator treatment were not observed. Trials are still in progress with completion expected by 2022.

With the varied effect on gene expression, depending on the chosen inhibitor and its cellular context, predicting the benefit of splicing modulation for a particular disease proves exceedingly difficult and necessitates the search for suitable prognostic markers. Perhaps, future promise lies in more specific, targeted inhibitors working on individual splicing factors, rather than throwing the entire pre-mRNA processing out of whack.

### New tools

**Fig. 4:**
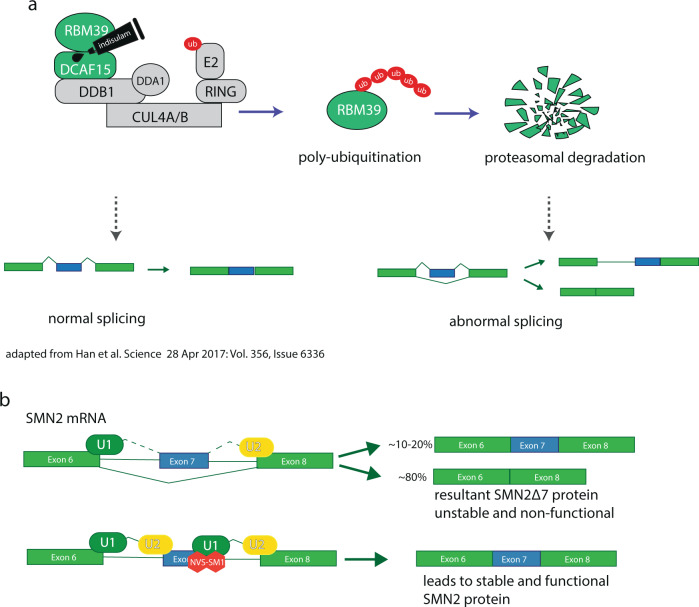
**a** Depiction of indisulam acting as a molecular glue, targeting RBM39 for proteasomal degradation. **b** Simplified depiction of the mechanism of branaplam (NVS-SM1) in increasing SMN2 exon 7 splicing efficiency. Risdiplam is thought to act in the same manner.

The identification of the sulfonamide anticancer drug indisulam points in a promising direction (Fig. 1c). Originally identified in a screen for molecules with antitumor activity [100, 101], indisulam appeared efficacious in xenograft models [102]. In clinical trials against solid tumors, indisulam elicited a response in some 10% of patients with no means of predicting which group of people treated would benefit from taking sulfonamides. Two independent research efforts, using a forward genetic [103] or proteomic approach [104] respectively, realized that indisulam and related sulfonamides E7820, tasisulam and CQS act as a molecular glue, tying the U2AF-related splicing factor RBM39 to DCAF15, an adaptor protein of the CUL4-DDB1-DDA1-DCAF15 E3 ubiquitin ligase complex (Fig. 4a). This association leads to RBM39 polyubiquitination and proteasome-dependent degradation. RBM39 has been identified as a coactivator for several transcription factors and a regulator of receptor-dependent alternative splicing, including splicing of VEGF [105, 106].

In absence of the sulfonamide DCAF15 and RBM39 have no affinity toward each other. Under indisulam treatment intron retention and ES were observed with only a few cases of changed 3ʹ and 5ʹ splice sites [103], as well as changes in the splice pattern of VEGF [104]. Mutational and structural studies identified a degron motif present in RBM39 and also RBM23, another U2AF-related splicing factor, which destined RBM23 and RBM39 to sulfonamide-dependent proteasomal degradation. In clinical phase II trials, about 1/3 of patients with chronic or relapsed acute myeloid leukemia (AML) responded to indisulam in combination with the nucleotide analogue cytarabine and DNA topoisomerase inhibitor idarubicin [107]. Perhaps, using the emerging targeted protein degradation technology to individually destroy a particular splicing factor holds promise in providing specific therapies with fewer side effects and more predictable clinical behavior.

### RNA-binding modulators

In addition, a further field of small molecule modulators in splicing has emerged, relying on compounds that primarily interact with RNA, not protein [108]. It was this area of investigation that led to the first clinically approved therapeutics altering splicing behavior to treat spinal muscular atrophy (SMA) [109].

Spinal muscular atrophy (SMA) is an autosomal recessive genetic disorder, occurring in about 1 out of 11,000 life births. A loss-of-function mutation in the survival of motor neurons 1 (SMN1) gene prevents cells from producing sufficient levels of SMN protein, essential for normal development and cellular homeostasis, not limited to motor neurons. A second gene, SMN2, is present in human cells, but a translationally silent C to T mutation at the 5ʹ end of exon 6 produces a weak splice site, leading to exon 7 exclusion. Consequently, only ~10% of functional SMN protein is produced, as resultant SMNΔ7 protein without exon 7 is unstable and inactive. Chemical screening for molecules enhancing splicing from a reporter gene containing the SMN2 exon6-intron7 splice site followed by optimization through medicinal chemistry led to NVS-SM1, the first molecule to specifically alter splicing behavior at a particular splice site (Fig. 1c). NVS-SM1, also labeled branaplam, enhances the interaction between the SMN2 pre-mRNA and the U1 snRNA, thereby turning a weak splice site into a strong one (Fig. 4b). While branaplam is still in clinical trials, three further therapies against SMA have gained FDA approval in recent years.

The first approved medication, nusinersen, relies on anti-sense oligonucleotides (ASO), and while efficacious, requires repeated intrathecal injections, making prolonged treatment expensive, risky and somewhat impractical. The nusinersen ASO acts by displacing the hnRNP A1/A2 splicing repressor from intron 7 of the SMN2 gene, enhancing exon 7 inclusion [110]. The second approved treatment, marketed as zolgensma, is a gene therapy, delivering functional SMN1 protein through an adeno-associated virus vector, aimed at infants under 2 years of age [111]. Branaplam phase 1 and 2 clinical trials were halted over toxicity concerns from an animal study but seem to have meanwhile resumed again (clinicaltrials.gov NCT02268552). At the beginning of 2021, branaplam did receive orphan drug status from the FDA, though as a treatment for Huntington’s disease. With branaplam trials halted over safety concerns, risdiplam, a competing compound became the first FDA approved oral medication for SMA. Likely sharing the same general mechanism with branaplam, risdiplam constitutes the result of extensive structural optimization, which seems to have yielded a molecule with higher specificity, less off-target toxicity and a more favorable pharmacokinetic profile [112].

While branaplam and risdiplam represent the best studied examples of compounds specifically changing splicing behavior at a particular exon, a recent report identified a small molecule, dubbed RECTAS, changing splicing behavior of the IKAP gene involved in familial dysautonomia, a hereditary sensory neuropathy [113]. Judging by its activity in vitro RECTAS may very well interact directly with the splicing or RNA-processing machinery. Recent findings indicated RECTAS’s potential in the treatment of Parkinson’s disease [114], however, its target still awaits discovery [115].

### Outlook

The discussed molecules constitute by no means the only way of pharmacologically changing splicing behavior. A whole body of research has evaluated the effects of CLK and DYRK kinase inhibitors on changing SR protein behavior and thereby alternative splicing. These signaling modulator show some promise in treating several diseases caused by underlying splicing defects, including Duchenne muscular dystrophy, familial dysautonomia and certain types of cystic fibrosis [116, 117]. A discussion of the role of signal transduction in splicing deserves its own review [118].

From a medicinal point of view, RNA specific medications and molecules exploiting the protein degradation machinery point in the future direction of chemical splicing modulation. This activity is not limited to mammalian mRNA processing, but promising results have also been obtained with molecules targeting group II self-splicing introns in pathogenic yeast [119], potentially providing a pathogen specific target to fight opportunistic *Candida parapsilosis* infections. While drug resistance should not become an issue in treating muscular atrophy, one needs to keep in mind that against cancer increasing specificity in mechanism also increases the chances of drug-specific resistance. Therefore, the potential of relatively broad inhibitors acting on novel targets should not be underestimated.

Splicing modulators provide great study objects for the promises and challenges in chemical biology. They display both the power of natural products as well as the necessity for medicinal chemistry in providing sufficient quantities of stable and potent molecular effectors. Furthermore, they demonstrate how structurally disparate molecules may share a binding site and mechanism of action, yet how small differences in binding may bring about very different cellular outcomes. With splicing modulators perturbing a fundamental process in gene expression control, the need to understand the cellular effects beyond the interaction between drug and target molecule becomes ever more apparent. Even that may not suffice when trying to understand the workings of a small molecule in an organismal context. The next years will show whether splicing modulators targeting SF3B will mature into efficacious therapeutics. In laboratory research, they have already proven themselves powerful bioprobes into the basis of gene expression and we can expect them to provide powerful tools for future discoveries.
